# Phylogenetic implications and characterization of the chloroplast genome of *Atractylodes macrocephala* (Compositae), an herb species to China

**DOI:** 10.1080/23802359.2019.1693306

**Published:** 2023-01-01

**Authors:** Bin Zhu, Tianyi Cao, Yuying Lang, Jun Fei

**Affiliations:** a Department of Pharmacy, Zhejiang Chinese Medicine and Western Medicine Integrated Hospital, Hangzhou, Zhejiang, People's Republic of China; b Zhejiang Integrated Traditional Chinese and Western Medicine Hospital, Hangzhou, Zhejiang, People's Republic of China

**Keywords:** Atractylodes macrocephala, Compositae, chloroplast genome, phylogenetic relationship, phylogenetic implications

## Abstract

The rhizome of *Atractylodes macrocephala* is one of the most commonly used herbs in China. In this paper, we presented the complete chloroplast genome of *A. macrocephala*. The chloroplast genome of *A. macrocephala* is 153,256 bp in length as the circular, which harbors a large single-copy (LSC) region 84,291 bp, a small single-copy (SSC) region of 18,675 bp and separated by a pair of inverted-repeat (IR) regions of 25,145 bp for each one. The overall nucleotide content of the chloroplast genome is 37.7% GC content. This chloroplast genome contains 125 genes, which includes 88 protein-coding genes (PCGs), 29 transfer RNA (tRNAs) and 8 ribosome RNA (rRNAs). Phylogenetic implications based on chloroplast genomes of 16 the family Compositae plant species indicated that *Atractylodes macrocephala* was closely related to *Atractylodes lancea* in the family Compositae by the Maximum-Likelihood (ML) method.

The rhizome of *Atractylodes macrocephala* (Bai-Zhu in Chinese) has been used in Traditional Chinese Medicine (TCM) for about 2,000 years in China (Shan 1981). It was awarded the title as the first herb of invigorating qi and strengthening spleen, which is also as one of eight well-known medicinal herb specialties in Zhejiang province and the most famous and much more expensive than other regions (Hu et al. 2019). The rhizome of *A. macrocephala* possesses various pharmacological effects that involves anti-mutation, anti-tumor, anti-aging, promotion of cellular growth and so on (Li et al. 2012). Now, the study of *A. macrocephala* mainly focuses on the composition and activity, little molecular biology and bioinformatics information about this species. In this paper, the chloroplast genome of *A. macrocephala* was presented that can provide some genome information and data for the family Compositae plant, also can be TCM business to the world in further.

The fresh root of *Atractylodes macrocephala* as the sample was collected from herb market near Zhejiang Chinese Medical University that located at Hangzhou, Zhejiang, China, 30.09 N, 119.89E. The chloroplast genomic DNA of *A. macrocephala* was extracted from the fresh root using the modified CTAB method and stored in Zhejiang Chinese Medical University (No. SCMC-ZJU-TCM-03). The NEB Next Ultra^TM^ II DNA Library Prep Kit (NEB, BJ, and CN) was used to purify the chloroplast DNA, which used by using sequencing. The low-quality reads and adapters was removed using FastQC software (Andrews 2015) for quality control. The chloroplast of *A. macrocephala* genome was assembled and annotated using the MitoZ software (Meng et al. 2019). The physical map of the chloroplast *A. macrocephala* genome used OGDRAW software (Greiner et al. 2019) to draw. The information and sequence of chloroplast genome had been deposited in GenBank with the accession number MN0446711.

The chloroplast genome of *Atractylodes macrocephala* is 153,256 bp in length which is the circular with a characteristic quadripartite structure. It harbors a large single-copy (LSC) region of 84,291 bp, a small single-copy (SSC) region of 18,675 bp and separated by a pair of inverted repeat (IR) regions of 25,145 bp each. The chloroplast genome of *A. macrocephala* contains 125 genes, which includes 88 protein-coding genes (PCGs), 29 transfer RNA genes (tRNAs) and 8 ribosomal RNA genes (rRNAs). In each IR region, the total of 17 genes, which included 8 PCG genes species (*rpl12, rpl23, ycf2, ndhB, rps, rps12, ycf15 a*nd *ycf1*), 5 tRNA genes species (*trnI-CAU, trnL-CAA, trnV-GAC, trnR-ACG* and *trnN-GUU*) and 4 rRNA genes species (*rrn16, rrn23, rrn4.5* and *rrn5*) were found duplicated. The overall nucleotide content of the chloroplast genome: 31.0% A (Adenine), 31.3% T (Thymine), 18.6% C (Cytosine), 19.1% G (Guanine), and 37.7% GC content.

To further investigate *Atractylodes macrocephala* phylogenetic implications, the Maximum-Likelihood (ML) was used to construct phylogenetic tree and based on chloroplast genomes of 16 the family Compositae plant species. ML phylogenetic tree analysis used the MEGA X software (Kumar et al. 2018) and performed using 2,000 bootstrap values replicate at each node. All of the nodes were inferred with strong support by the ML methods. The final NJ phylogenetic tree was edited using the iTOL version 4.0 (https://itol.embl.de/) (Letunic and Bork 2016). Phylogenetic implications indicated that *Atractylodes macrocephala* was closely related to *Atractylodes lancea* in phylogenetic relationship (Figure 1). This paper can provide much information and data for the family Compositae plant and more can be TCM business to the world.

**Figure 1:**
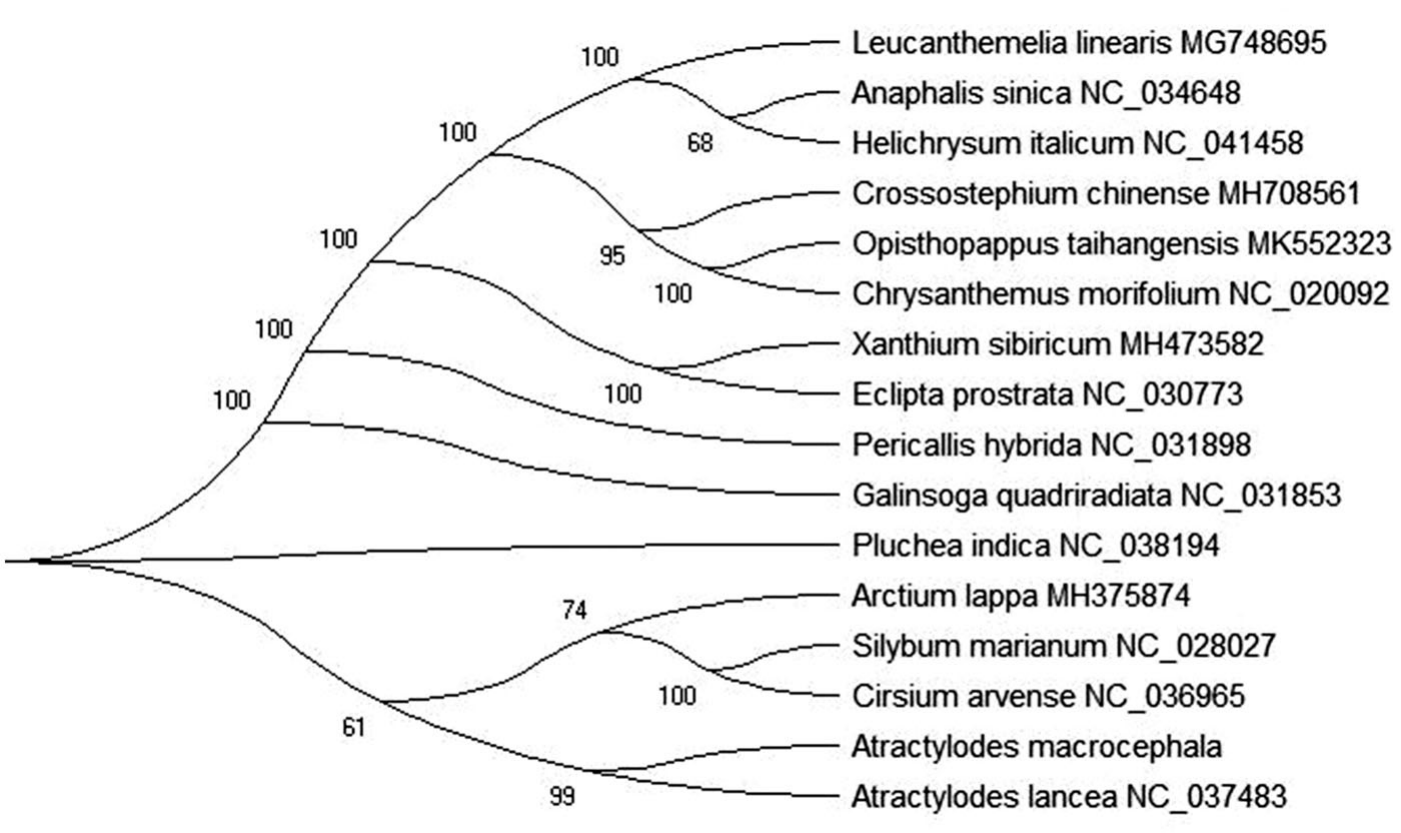
Maximum-likelihood phylogenetic tree based on the complete chloroplast genome sequences of 17 the family Compositae plant species. Bootstrap support values based on 2,000 replicates are shown next to the nodes for each branch.
